# 

**Published:** 1899-09-02

**Authors:** 


### NOTICE TO CORRESPONDENTS.

All MS., Letters, Books for Review, and other matters intended for the Editor should be addressed The Editor, "The Hospital," The Hospital Building, 28 & 29, Southampton Street, Strand, W.C.

The Editor cannot undertake to return rejected MS., even when accompanied by stamped directed envelope.

**Notice to Advertisers and Subscribers.**

All Advertisements, Orders for Copies of The Hospital, and Business Communications should be addressed to **THE MANAGER (Not to the Editor)**, The Hospital, The Hospital Building, 28 & 29, Southampton Street, Strand, London, W.C.

The Cost of Subscription, post free, is as follows:—Per week, 2½d.; per quarter, 2s. 8d.; per half-year, 5s. 3d.; per annum, 10s. 6d. Foreign:—Per annum, 15s. 2d., payable, in all cases, in advance.

**NOTICE.—Vol. XXV. of "The Hospital" (October, 1898, to March, 1899), in handsome cloth binding, is now on sale at the Office of this Journal, price 7s. 6d. Cases for binding the Numbers contained in this Volume 1s. 6d. Index to Vol. XXV., 2½d., post free.**

**The Monthly Part for September ie now ready. Price 6d., by post 8d.**

### BOOKS RECEIVED.

J. and A. Churchill.

"Notes on Folkestone." By Arthur E. Larking, M.D.

"The Open-air Treatment of Phthisis." By W. Bezly Thorne, M.D., M.R.C.P.

The Scientific Press.

"Nursing: Its Theory and Practice." By Percy G. Lewis, M.D. Thirteenth thousand.

Dotesio and Todd (Lowestoft).

"Lowestoft as a Winter Resort."

John Wright and Co.

"Golden Rules of Psychiatry." By Jas. Shaw, M.D.

"Annuaire Statisque de la Ville de Buénos Ayres."

